# Assessing cardiovascular risk in regional areas: the Healthy Hearts – Beyond City Limits program

**DOI:** 10.1186/1472-6963-12-296

**Published:** 2012-09-03

**Authors:** Melinda J Carrington, Garry L Jennings, Robyn A Clark, Simon Stewart

**Affiliations:** 1 Baker IDI Heart and Diabetes Institute, 75 Commercial Rd, Melbourne, Vic, 3004, Australia; 2 School of Nursing and Midwifery, Queensland University of Technology, Queensland, Ring Rd, Kelvin Grove Campus, Kelvin Grove, , QLD, 4059, Australia

**Keywords:** Cardiovascular disease, Prevention, Risk assessment, Risk factors

## Abstract

**Background:**

Cardiovascular disease (CVD) is more prevalent in regional and remote Australia compared to metropolitan areas. The aim of Healthy Hearts was to determine age and sex specific CVD risk factor levels and the potential value of national risk clinics.

**Methods:**

Healthy Hearts was an observational research study conducted in four purposefully selected higher risk communities in regional Victoria, Australia. The main outcome measures were the proportion of participants with CVD risk factors with group comparisons to determine the adjusted likelihood of elevated risk factor levels. Trained personnel used a standardized protocol over four weeks per community to measure CVD risk factor levels, estimate absolute CVD risk and provide feedback and advice.

**Results:**

A total of 2125 self-selected participants were assessed (mean age 58 ± 15 years, 57% women). Overall, CVD risk factors were highly prevalent. More men than women had ≥ 2 modifiable CVD risk factors (76% vs. 68%, p < .001), pre-existing CVD (20 vs. 15%, p < .01) and a major ECG abnormality requiring follow-up (15% vs. 7%, p < .001) . Less men reported depressive symptoms compared to women (28% vs. 22%, p < .01). A higher proportion of women were obese (adjusted OR 1.36, 95% CI 1.13 to 1.63), and physically inactive (adjusted OR 1.32, 95% CI 1.07 to 1.63).

**Conclusions:**

High CVD risk factor levels were confirmed for regional Victoria. Close engagement with individuals and communities provides scope for the application of regional risk management clinics to reduce the burden of CVD risk in regional Australia.

## Background

Amid improvements in cardiovascular disease (CVD) related mortality, risk factors remain high in adult Australians. Age-adjusted case fatality rates from CVD have fallen from 55% of all deaths in the late 1960s to 34% in 2007 [1]. This is attributable to better CVD prevention, detection and clinical management. Nevertheless, CVD (notably coronary heart disease) ranks second highest in healthy years of life lost, representing 16% of the overall disease burden [1]. Inevitably, the burden of CVD will inexorably rise within Australia’s ageing population [2]. Despite some encouraging trends in respect to the declining prevalence of major risk factors [1,3], there are equally cautionary data in respect to sustained, and in some cases increasing levels of hypertension [4,5], dyslipidaemia [1,6] and metabolic risk factors (e.g. physical inactivity/sedentary behavior[1] and obesity [3,7]). When coupled with increasing longevity, it is therefore imperative that heart health be improved by mitigating elevated risk factors [8] through pharmacological and/or non-pharmacological recommendations embedded within evidence-based guidelines.

Any focus on reducing cardiovascular risk levels has to consider higher risk populations. People living in regional, rural or remote locations consistently fare worse than their metropolitan counterparts [5,9,10] with mortality rates rising in accord with remoteness [1]. Unfortunately, reliable (measured) population data, especially for biomedical health risk factors, are over 10 years old and limited in respect to regional data. This extends to the AusDiab Study and local Crossroads Undiagnosed Disease Study. Therefore, the overall aim of the *Healthy Hearts Beyond City Limits* program was two-fold; firstly to confirm elevated levels of cardiovascular risk in key regional Victorian communities according to age and sex and to simultaneously determine the scope to engage such communities and individuals to reduce elevated levels of risk (if confirmed) through regional risk management clinics.

## Methods

### Study setting and design

**Figure 1:**
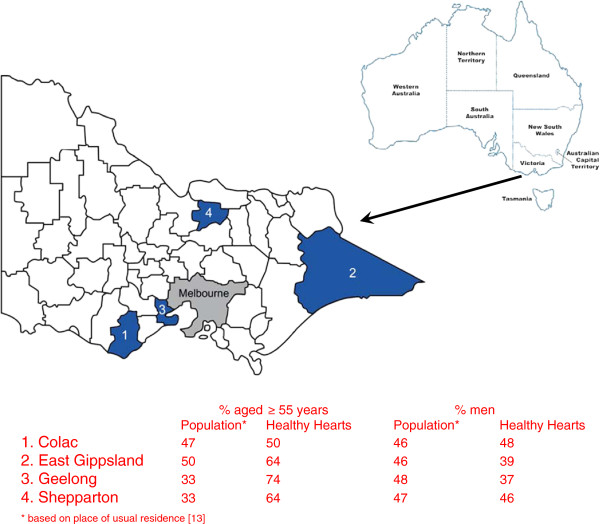
Local government areas identifying four target regional communities of Victoria.

*Healthy Hearts* was a regional observational research study. In order to distribute limited resources to areas that required it most, we used Geographical Information System profiling [11] to identify regional areas with >20,000 total population in Victoria and which had an increased prevalence of chronic heart failure, Aboriginal and Torres Strait Islanders, obese children aged 7–18 years and adults over 65 years of age. Of 10 high risk communities identified, four relatively geographically dispersed were purposefully selected to visit (see Figure 1). These included Colac (adult population of 7,172 [based on place of usual residence] and 152 km South-West of Melbourne), East Gippsland (11,251 and 294 km North-East of Melbourne), Geelong (3,664 and 75 km South-West of Melbourne) and Shepparton (20,410 and 190 km North of Melbourne). 

Co-ordination of the *Healthy Hearts* program was facilitated by the local Rotary Clubs in each community. The program was operational between 0830 and 1700 on all weekdays. We had the capacity for approximately 40 assessments per day, averaging five per hour or 12 minutes per participant. A combination of free heart health checks via a mobile risk assessment unit were undertaken in public settings such as a shop or park location (73% of all assessments) or via dedicated workplace visits to key businesses. Assessments were performed concurrently by a team of at least four Registered Nurses and fully trained research personnel according to a standardized protocol. The study was approved by the Human Research Ethics Committee at the Alfred Hospital, Melbourne, Australia (Project No. 71/07) and the STROBE guidelines were referred to in reporting studies of this nature [12].

### Participants

We aimed to conduct at least 500 health checks over a continuous 3–4 weeks of screening per community in 2007–2010. Prior to our arrival, the program was advertised in the local newspaper(s) and radio station and key businesses were notified. Participants self-selected and the only inclusion criteria were to be over 18 years of age with the ability to provide written consent to participate. Overall, 2,125 participants from the four regional communities volunteered to have a risk assessment. The proportion of the adult population assessed (based on place of usual residence) was 8% in Colac, 5% in East Gippsland, 14% in Geelong and 3% in Shepparton [13].

### Data collection

The program comprised three stages; 1) self-report questionnaire, 2) non-invasive clinical assessment and, 3) absolute CVD risk assessment report and brief consultative review. The self-administered questionnaire incorporated validated assessment instruments and examined the following: socio-demographic indicators; diet and lifestyle habits such as smoking (current smoker, ex smoker or never smoked); fat intake using the MEDFICTS dietary assessment tool [14]; physical activity via the International Physical Activity Questionnaire [15]; personal and family medical history; medication use; mental health via the 2-item Arroll questionnaire [16] and CES-D [17]; angina and intermittent claudication symptoms using the Rose Angina questionnaire [18]; and overall health and well-being via the SF-12 [19]. Education was classified as either secondary school or below or higher than secondary school. Clinical assessments included measurement of blood pressure (BP), height, weight, anthropometric measurements of abdominal and hip circumference, point of care random lipid and glucose profiling, electrocardiography (ECG), spirometry, and in a sub-sample, the ankle brachial pressure index to identify peripheral arterial disease. The results from many of these variables will be the focus of a number of future publications. In the final part of participation, a summary report describing an individual’s cardiovascular risk factor profile, 5-year (primary) [20,21] or 2-year (secondary) [22] absolute CVD risk score and 5-year type 2 diabetes risk [23] was given to participants and the details were explained by a senior member of the *Healthy Hearts* team (MC, SS or senior cardiac nurse). During the feedback session, advice and education was given to address any risk factors; in the case of an extreme or adverse test result, participants were advised to consult their general practitioner (GP) for follow-up or were directed to hospital for more urgent cases.

#### Instrumentation and procedures

After 5 minutes of rest, BP in the brachial artery was measured using a validated digital BP monitor (Dinamap® PROCARE 300, GE Healthcare, Buckinghamshire, UK) [24] in the sitting position with an appropriately sized cuff and table support for the measured arm. The average of two measurements separated by a one-minute interval was analyzed provided there were no large variations in systolic (≥10 mmHg) or diastolic (≥7 mmHg) BP, in which case another reading was taken and the closest two readings were analyzed. Height and weight for assessing body mass index (BMI, kg/m^2^) were measured using a stadiometer and digital weighing scales, with the removal of shoes and heavy garments. Abdominal and hip circumference were measured in the horizontal plane whilst standing, in accord with the World Health Organization (WHO) Stepwise approach to surveillance (STEPS) procedure [25]; the level mid-way between the lowest rib and iliac crest at the end of a gentle expiration was taken for abdominal circumference and the level at the maximum extension of the buttocks defined hip circumference. Lipid and glucose measurements were analyzed by a validated Cholestech LDX® System (Cholestech Corporation, CA, USA) [26,27]. Portable PC-based 12-lead ECGs (Universal ECG™) were collected in adherence with standard electrode placement using Office Medic™ Software (QRS Diagnostic, MN, USA). Absolute cardiovascular risk for primary prevention [21] was calculated using age, gender, smoking status, diabetes, systolic BP and total cholesterol (TC)/high density lipoprotein cholesterol (HDL-C) ratio, with an adjustment for extremely elevated TC, diastolic BP, kidney disease and type 2 diabetes [20]. The variables for secondary prevention were age, gender, diabetes, TC, HDL-C and for women only, systolic BP and smoking status[22].

#### Risk factor definitions

Ideal individualized risk factor targets were based on national guidelines, reports and validated assessment tools. Those who currently smoked or had stopped smoking less than 12 months before the assessment were defined as a smoker. Energy intake from dietary saturated fat scores were classified as low (<40), moderate (40–69) or high (≥70) [14]. Physical inactivity was defined as less than 150 minutes per week of activity [28], with vigorous intensity activity counted as double time and moderate intensity or brisk walking counted as single time [1]. Excess alcohol consumption was defined as >2 standard drinks on any day [29]. Participants who answered *yes* to either of two screening questions for potential depression [16] proceeded to complete the CES-D whereby depression was indicated by a score of ≥16 [17].

Hypertension was defined as BP ≥ 130/80 mmHg for participants with associated condition(s) or end organ damage (n = 517), and for all others as BP ≥ 140/90 mmHg[30]. Participants were classified by BMI as normal weight (< 25 kg/m^2^), overweight (25 - < 30 kg/m^2^) or obese (≥ 30 kg/m^2^) [31]. For abdominal circumference, normal weight was classified as <94 cm for men and <80 cm for women, overweight for measurements between 94 to <101.9 cm (men) and 80 to <87.9 cm (women), and obese for measurements ≥102 cm for men and ≥88 cm for women [1]. For higher risk participants taking lipid-modifying therapy (n = 395), treatment target levels used to determine optimal lipid levels were <4 mmol/L for TC, <2.0 mmol/L for low density lipoprotein cholesterol (LDL-C) and <1.5 mmol/L for triglycerides [32]. The recommended target levels for all other participants not on lipid-modification treatment were 5.5 mmol/L for TC, 3.0 mmol/L for LDL-C and 2.0 mmol/L for triglycerides [1,7]. For all participants, the target level for HDL-C was 1.0 mmol/L [32] and <6.9 or <11.0 mmol/L for fasting and random glucose levels, respectively [33]. Dyslipidaemia was defined as not meeting any one of the lipid target levels just described. Metabolic syndrome was defined by abdominal obesity in the presence of any of two, including treatment for, the following deficiencies: raised levels of triglycerides, BP, fasting glucose (where applicable) or reduced HDL-C [34]. Absolute primary and secondary CVD risk was classified as low (≤9%), moderate (10-15%) or high (≥16%), defaulting those with extremely elevated TC or BP, kidney disease, or type 2 diabetes at >60 years of age to high risk (primary prevention risk scores only) [20]. All ECGs were systematically scored by a trained cardiac nurse using the Minnesota Code [35] and confirmed by SS.

### Data analyses

Data were analyzed using SPSS Statistics 19.0. Normally distributed continuous data are presented as the mean ± standard deviation and non-Gaussian distributed variables as the median plus interquartile range. Categorical data are presented as percentages. Discrete variables were analyzed via odds ratios (OR) with 95% confidence intervals (CI) or *χ*^2^ analysis. Students t-tests were used for continuous variables with consideration to Levene’s Test for Equality of Variances to correct for any violations of the assumption of homogeneity of variance. Multiple logistic regression analyses (entry model) were performed on age, sex, community, education and potential depression to derive adjusted ORs for the likelihood estimates of CVD risk factors. Significance was accepted at the two-sided level of 0.05.

## Results

### Study cohort

able 1 summarizes the socio-demographic and clinical risk factor profiles according to sex. There were more women (1218, 57%) than men who were of a similar age and both sexes comprised mostly people from a Caucasian/European ethnic background (96%; OR 0.88, 95% CI 0.58-1.32). More men than women were married/living with a partner (OR 1.92, 95% CI 1.56-2.36) whilst less men had received maximum secondary school education (OR 0.59, 95% CI 0.49-0.70). More than half were currently employed unskilled workers and one quarter were employed in a professional role with more male technical/trades people and more unskilled female workers.

**Table 1:** Socio-demographic and risk profile of participants

	**All n = 2125**	**Men n = 907 (43%)**	**Women n = 1218 (57%)**	**OR [95% CI]**	***p*value**
***Socio-demographic profile***					
Age (years)	57.6 ± 14.6	57.2 ± 15.2	58.0 ± 14.2		.209
Male gender	907 (43%)	907 (100%)			—
Caucasian ethnicity	2030 (96%)	863 (95%)	1167 (96%)	0.88 [0.58-1.32]	.523
Married / living with partner	1566 (74%)	727 (81%)	839 (69%)	1.92 [1.56-2.36]	<.001
Secondary school highest education	1203 (59%)	452 (38%)	751 (62%)	0.59 [0.49-0.70]	<.001
Current occupation:					
Professionals/Semi-professionals	251 (25%)	123 (26%)	128 (25%)		<.001
Technical/Tradespeople	191 (19%)	137 (29%)	54 (10%)		
Unskilled workers	559 (56%)	220 (46%)	339 (65%)		
***Risk factor profile***					
Current smoking	227 (11%)	104 (12%)	123 (10%)	1.15 [0.87-1.52]	.313
Systolic blood pressure (mmHg)	137 ± 21	141 ± 19	134 ± 22		<.001
Diastolic blood pressure (mmHg)	75 ± 11	79 ± 10	73 ± 10		<.001
Body mass index (kg/m^2^)	27.7 ± 5.1	28.0 ± 4.5	27.5 ± 5.5		.035
Total cholesterol (mmol/L)	5.21 ± 1.13	5.14 ± 1.17	5.26 ± 1.10		.013
LDL-C (mmol/L)	2.98 ± 1.02	3.02 ± 1.04	2.96 ± 1.00		.175
HDL-C (mmol/L)	1.34 ± 0.43	1.16 ± 0.38	1.47 ± 0.42		<.001
TC/HDL ratio (mmol/L)	4.22 ± 1.70	4.77 ± 1.89	3.81 ± 1.42		<.001
Glucose (mmol/L)	5.80 ± 1.53	6.02 ± 1.72	5.64 ± 1.36		<.001
Alcohol consumption (drinks per week)	4.3 ± 7.8	6.51 ± 10.28	2.85 ± 5.02		<.001
Physical activity (MET-minutes per week)	998 [446–2083]	1118 [518–2611]	924 [396–1817]		<.001
Absolute COD risk (%)					
Primary	8 ± 6	12 ± 7	6 ± 4		<.001
Secondary	6 ± 4	9 ± 2	3 ± 2		<.001
≥ 2 modifiable risk factors	1508 (71%)	686 (76%)	822 (68%)	1.50 [1.23-1.81]	<.001
Family history of CVD	673 (32%)	266 (29%)	407 (33%)	0.83 [0.69-1.00]	.045
Pre-existing CVD	360 (17%)	178 (20%)	182 (15%)	1.39 [1.11-1.75]	.004
Type 2 diabetes/hyperglycaemia	124 (6%)	60 (7%)	64 (5%)	1.28 [0.89-1.85]	.178
Potential depression	514 (25%)	190 (22%)	324 (28%)	0.72 [0.59-0.89]	.002
Major ECG abnormality	209 (11%)	129 (15%)	80 (7%)	2.35 [1.75-3.16]	<.001

*LDL-C*: low density lipoprotein; *HDL-C*: high density lipoprotein; *MET*: metabolic equivalent of task; *CVD*: cardiovascular disease; *mmHg*: millimeters of mercury; mmol/L: millimols per liter.

**Figure 2:**
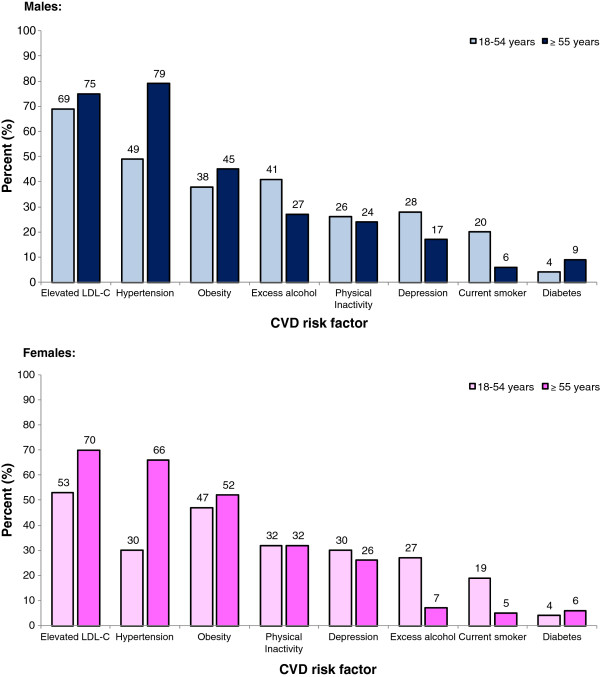
Proportion of participants with elevated risk factors according to sex and age.

Overall, CVD risk factors were highly prevalent (Table 2). Regardless of gender, approximately two thirds of participants had elevated TC or LDL-C, 59% hypertension and 28% obesity by BMI. A total of 29% were physically inactive, 6% had diabetes/hyperglycaemia, 21% reported increased alcohol consumption, 11% were smokers and 25% had potential depression. Figure 2 highlights the proportion of men and women by age group who were assessed with a cardiovascular risk factor(s). The three most prevalent risks were elevated LDL-C, hypertension and obesity. Older adults (aged ≥ 55 years) had more elevated LDL-C, hypertension, obesity (either by BMI or waist circumference) and diabetes, whereas a greater proportion of younger adults (aged 18 to 54 years) were smokers and reported increased alcohol consumption and depression. A similar proportion of older and younger adult men and women were physically inactive.

**Table 2:** Proportion of participants with risk factors according to sex and region

	**Colac**		**East Gippsland**		**Geelong**		**Shepparton**		**All**
	**n = 548 (26%)**		**n = 552 (26%)**		**n = 520 (24%)**		**n = 505 (24%)**		
	**Men**	**Women**	**Men**	**Women**	**Men**	**Women**	**Men**	**Women**	
	**n = 265**	**n = 283**	**n = 218**	**n = 334**	**n = 191**	**n = 329**	**n = 233**	**n = 272**	
***Socio-demographic risk factors:***									
Aged over 55 years	123 (46%)	149 (53%)	146 (67%)	207 (62%)	144 (75%)	241 (73%)	144 (62%)	178 (65%)	1,332 (63%)
Male gender (% of total cohort)	265 (48%)		218 (40%)		191 (37%)		233 (46%)		907 (43%)
***Risk factor profile:***									
Current smoking	38 (14%)	27 (10%)	25 (12%)	39 (12%)	11 (6%)	23 (7%)	30 (13%)	34 (13%)	227 (11%)
Hypertension	179 (68%)	158 (56%)	155 (71%)	178 (53%)	113 (59%)	149 (45%)	161 (69%)	157 (58%)	1,250 (59%)
Obese (BMI)	59 (22%)	71 (25%)	49 (23%)	90 (27%)	37 (19%)	84 (26%)	109 (47%)	96 (35%)	595 (28%)
Abdominal obesity	92 (35%)	118 (42%)	69 (32%)	173 (52%)	68 (36%)	170 (52%)	125 (54%)	137 (51%)	952 (45%)
Elevated total cholesterol	199 (76%)	192 (69%)	147 (71%)	210 (65%)	111 (58%)	194 (59%)	148 (64%)	157 (58%)	1,358 (65%)
Elevated LDL cholesterol	194 (85%)	190 (72%)	143 (75%)	191 (65%)	119 (67%)	189 (61%)	129 (63%)	147 (57%)	1,302 (67%)
Reduced HDL cholesterol	86 (34%)	33 (12%)	60 (29%)	36 (11%)	80 (43%)	39 (12%)	75 (33%)	27 (10%)	436 (21%)
Metabolic syndrome	113 (43%)	97 (34%)	93 (43%)	133 (40%)	85 (45%)	130 (40%)	135 (58%)	114 (42%)	900 (42%)
Increased alcohol consumption	—	—	66 (30%)	56 (17%)	46 (24%)	29 (9%)	91 (39%)	41 (15%)	329 (21%)
Physical inactivity	66 (27%)	91 (35%)	34 (17%)	99 (33%)	45 (25%)	82 (26%)	60 (29%)	83 (34%)	560 (29%)
Absolute CVD risk (primary & secondary):									
Low risk	65 (44%)	136 (84%)	68 (46%)	195 (85%)	62 (48%)	209 (89%)	74 (49%)	158 (86%)	967 (70%)
Moderate risk	49 (33%)	19 (12%)	48 (33%)	28 (12%)	46 (36%)	24 (10%)	47 (31%)	22 (12%)	283 (20%)
High risk	34 (23%)	7 (4%)	31 (21%)	6 (3%)	21 (16%)	1 (1%)	31 (20%)	4 (2%)	135 (10%)
≥ 2 modifiable risk factors	202 (76%)	194 (69%)	164 (75%)	233 (70%)	132 (69%)	201 (61%)	188 (81%)	194 (71%)	1,508 (71%)
Family history of CVD	55 (21%)	68 (24%)	49 (23%)	103 (31%)	75 (39%)	125 (38%)	87 (37%)	111 (41%)	673 (32%)
Pre-existing CVD	49 (19%)	47 (17%)	52 (24%)	46 (14%)	31 (16%)	41 (13%)	46 (20%)	48 (18%)	360 (17%)
Type 2 diabetes/hyperglycaemia	18 (7%)	13 (5%)	13 (6%)	20 (6%)	11 (6%)	11 (3%)	18 (8%)	20 (7%)	124 (6%)
Potential depression	42 (16%)	56 (21%)	30 (14%)	83 (26%)	45 (24%)	108 (34%)	73 (33%)	77 (30%)	514 (25%)

LDL cholesterol calculated for 1,930 participants, physical inactivity available for 1,949 and absolute CVD risk estimated for 1,385 cases.

### Sex-based differences

Table 1 shows that there were no sex-based differences in the proportion of current smokers (OR 1.15, 95% CI 0.87-1.52), LDL-C levels (*p >* .05) or type 2 diabetes/hyperglycaemia (OR 1.28, 95% CI 0.89-1.85). On average, men had 7 mmHg and 6 mmHg higher systolic and diastolic BP (both *p* < .001) and were borderline hypertensive. Men were more overweight according to BMI with 75% classified as either overweight or obese compared to 65% of women (data not shown) and had significantly higher glucose levels, consumed over twice the number of standard drinks of alcohol/week and were more physically active than women. Conversely, women had higher TC levels but also higher HDL-C levels and a lower TC to HDL-C ratio (denoting reduced risk). Average CVD risk scores were twice and three times as high in men for primary and secondary prevention, respectively. More men than women had ≥2 modifiable risk factors (OR 1.50 95% CI 1.23-1.81) and a prior history of the same (OR 1.39, 95% CI 1.11-1.75). Alternatively, less men reported depressive symptoms (OR 0.72, 95% CI 0.59-0.89). Of those who had an ECG as part of their assessment (1951, 92%), 209 (11%) participants had a potentially significant abnormality with two-fold more GP referrals in men (OR 2.35, 95% CI 1.75 to 3.16).

### Regional comparisons

Table 2 indicates the (unadjusted) number of participants with risk factors according to sex and region. More women (approximately two thirds) from East Gippsland and Geelong participated compared to the other two communities which had a greater gender balance. The age profile of men and women was similar across all communities. There were also similarities between men and women in smoking status and type 2 diabetes/hyperglycaemia (although two-fold more men in Geelong presented with the latter). The proportion of men in all communities with hypertension, elevated TC and LDL-C, reduced HDL-C and increased alcohol consumption was greater than women. Typically, more women were physically inactive and indicated a positive family history of CVD. Excepting Shepparton, more women were obese according to both BMI and abdominal circumference and reported depressive symptoms. Overall, more men than women were determined to have the metabolic syndrome, ≥2 modifiable risk factors, pre-existing CVD or higher estimated absolute CVD risk levels in the moderate (33% vs. 11%) and high risk categories (20% vs. 2%).

### Correlates of elevated risk

The multiple logistic regression models that we specified correctly predicted between 58% and 84% of individuals with CVD risk factors. On an adjusted basis, the odds for CVD risk were lower in women than men, ranging from an OR 0.31 (95% CI 0.24 to 0.42) for increased alcohol consumption to OR 0.82 (95% CI 0.67 to 1.0) for elevated TC. Conversely, women were more likely to be obese (OR 1.36, 95% CI 1.13 to 1.63) and physically inactive (OR 1.32, 95% CI 1.07 to 1.63). For each additional year of age, the odds significantly increased from a range of 2% (for LDL-C) to 7% (for hypertension) [95% CI range 1.01-1.06 to 1.02-1.08] but decreased by 4% (95% CI 0.95 to 0.97) for increased alcohol consumption. Lower education level was associated with greater CVD risk (adjusted OR range 1.25 to 1.55, 95% CI range 1.02-1.28 to 1.53-1.88). Generally, the odds for CVD risk factors were significantly lower for Geelong residents compared to Colac (adjusted OR range 0.37 to 0.56, 95% CI range 0.27-0.39 to 0.49-0.80) and for those without depressive symptoms (adjusted OR range 0.68 to 0.80, 95% CI range 0.50-0.64 to 0.92-0.98). Shepparton residents were more likely to be obese (adjusted OR 1.70, 95% CI 1.31 to 2.21) and have increased alcohol consumption compared to Geelong residents (adjusted OR 1.74, 95% CI 1.23 to 2.47).

## Discussion

The *Healthy Hearts Beyond City Limits* program, involving over 2,000 adults from four diverse regional locations, is the largest surveillance study of CVD-related risk factors and associated lifestyle and health behaviors utilizing measured results in regional Victoria, Australia. Overall, high levels of risk factors for CVD were common in all age groups. The proportion of participants outside individualized recommended levels ranged from 68% for elevated LDL-C to 11% for smoking, with abnormal cholesterol levels, raised BP and excess weight affecting half of participants. Younger adults more frequently reported smoking and increased alcohol consumption as well as depressive symptoms, the latter being more evident in women from most regional communities. Independent of regional location, men lagged behind women in many CVD risk indicators. Alternatively, more women were obese and physically inactive. Inter-community differences were evident with participants from the larger community and surrounding areas of Geelong having more favorable risk profiles compared to Colac who had the worst, yet those living in Shepparton were more obese and had excess alcohol consumption. High levels of risk confirmed a persistent problem in these communities. Alternatively, high levels of engagement reaffirmed the potential value of an extended program combining surveillance and active prevention.

The unadjusted prevalence of elevated BP, TC and LDL-C observed in this cohort was substantially higher than previous reports [9,10,36,37] but probably reflects participant self-selection and application of individualized (treatment) targets. Our older study cohort may partially, although not completely, explain the higher proportion of hypertension found in the rural Greater Green Triangle Risk Factor Study [37]. Alternatively, it is possible that BP levels are rising [5]. In the absence of (representative) population data, it is difficult to truly quantify the problem of elevated BP in regional Victorians. The (predominantly urban) AusDiab study found a lower prevalence of elevated TC [9] based on a cut-off of 5.5 mmol/L, but in the absence of regional differentials, there are no recent population data to compare our findings to assess whether they parallel national declining trends in primary care [6] and indeed global patterns [38]. Equally concerning were the high rates of overweight and obesity, a trend now seen world-wide [39]. Compared to contemporary regional estimates (78% for men and 52% for women) [3], our findings were slightly lower for men yet higher for women and generally overestimated compared to state statistics [10]. However, other studies have relied upon self-reported and therefore underestimated BMI [40]. Of concern, one in four adults reported depression. This appeared higher than the latest population estimates but confirmed that women were more likely affected than men [9]. In the latest National Health Survey, regional Australians were 16% more likely to report a mental health problem [41].

Despite highly prevalent CVD risk factors, absolute CVD risk scores were not congruently elevated emphasizing the well known limitation of risk classification for “low risk” who demonstrate coronary atherosclerosis [42]. Unfortunately, the introduction of new biomarkers [43] and non-invasive tests [42] are yet to improve the predictive ability of risk equations in asymptomatic individuals from a practical perspective. The end point of what to predict (e.g. CVD or coronary heart disease) also remains vague [42] yet Australian guidelines advocate for a composite CVD risk score [20] to identify individuals who may benefit from preventative therapy. All participants in the *Healthy Hearts* program, regardless of absolute CVD risk levels, were counseled on recommended risk factors levels and ideal diet and lifestyle behaviors or pharmacological treatment (if necessary). Exploring individuals’ present risk factors and future CVD risk was highly engaging with strong potential for significant risk reduction at earlier stages across the spectrum of CVD.

An individual’s risk of illness cannot be considered in isolation from their wider community. We observed heterogeneity in respect to the risk profiles of individuals in the different communities. This undoubtedly reflects the differentials of the socio-demographic profiles of participants but also reflects the differences in the characteristics of the broader communities in which these individuals lived. This includes the size, geographic location and level of local health care services. At the individual level, older communities will likely have elevated BP levels, and places with reduced access to a healthier and cheaper food supply might have higher levels of metabolic disturbances. As such there is likely to be considerable heterogeneity in the specific risk profile and health care needs of regional communities, requiring adaptation of services at their local level. Overall, Australia’s ageing population has resulted in significant retirement migration from urban to regional areas. Simultaneously, the closure or down-grading of local hospitals to aged care centers has created a mismatch between the supply and demand for regional health care services. Specialist cardiac services are scarce and few cardiologists practice in non-metropolitan areas [41]. The burden of health care is predominantly transferred to regional primary care services [41] where there are 87 full time equivalent GPs/100,000 population compared to 98 in major cities [44]. Financially stimulated growth in regional GP numbers has been offset by reduced operational hours [44]. Ominously, regional Australians are more likely to die from ischaemic heart disease (44%) or stroke (31%) than those living in major cities [41] with even worse differentials for fatal hypertensive heart disease (90% more likely) and heart failure (70%) events.

These data underlie the potential value of regional risk clinics to support already stressed primary care services. Encouragingly, the degree of individual and community engagement in the *Healthy Hearts* program exceeded expectations. Participating individuals overcame barriers to limited access to health care that is typical of regional community life and this program provides the impetus to establish cost-effective community-based risk management clinics. This was the focus of the soon to be reported Protecting *Healthy Hearts* program undertaken in regional Victoria, Australia.

There are a number of important limitations which may influence the interpretation and generalizability of our findings. Given limited resources we selected higher risk communities and participants were self-selected resulting in age and gender differences compared to population estimates in some communities (refer Figure 1). Self-selection potentially introduced further bias towards those with higher levels of risk, albeit the proportion of smokers (11%) we found was reduced compared to national population surveys (18%) [1], possibly due to our older cohort and the trend of decreased smoking prevalence with age. We measured lipid profiles in the non-fasting state in many participants (93%) based on findings that levels of TC, LDL-C, HDL-C, TC to HDL-C ratio and triglycerides are minimally affected by normal food intake in individuals in the general population [45]. It is also unlikely that the lipid profile response to typical food intake would vary according to sample timing. We cannot discount seasonal weather confounders and extraneous factors such as drought and floods which might particularly affect farmers.

## Conclusions

In summary, the *Healthy Hearts* program confirmed a high proportion of individuals with elevated CVD-related risk factors seeking to better understand their heart health. These data suggest that more needs to be done for regional Australian adults to address inequitable rates (short to longer term) of CVD relative to their metropolitan counterparts. The overwhelming positive response towards the *Healthy Hearts* program shows there is scope to engage communities and individuals to reduce elevated levels of risk and underlies the potential value of risk management clinics and nurse-led intervention programs in regional areas.

## Competing interests

The authors declare that they have no competing interests.

## Authors’ contributions

MC participated in the design, co-ordination and data collection of the study, carried out the analyses and interpretation of the data and drafted the manuscript. GJ participated in the conception and design of the study. RC participated in the design of the study. SS conceived of the study, participated in the design, co-ordination and data collection and contributed to analyses and interpretation of the data. GJ, RC and SS were involved in revising the manuscript critically for important intellectual content. All authors read and approved the final manuscript.

## Pre-publication history

The pre-publication history for this paper can be accessed here:

http://www.biomedcentral.com/1472-6963/12/296/prepub
